# The DNase TREX1 is a substrate of the intramembrane protease SPP with implications for disease pathogenesis

**DOI:** 10.1007/s00018-025-05645-5

**Published:** 2025-03-12

**Authors:** Onur Kerem Tever, Torben Mentrup, Ivan Kingyue Chinn, Hitoshi Ishikuma, Regina Fluhrer, Marc Schmitz, Rebekka Wehner, Rayk Behrendt, Javier Chinen, Bernd Schröder

**Affiliations:** 1 Institute for Physiological Chemistry, Faculty of Medicine Carl Gustav Carus, Medizinisch-Theoretisches Zentrum MTZ, Technische Universität Dresden, Fiedlerstraße 42, 01307 Dresden, Germany; 2 Department of Pediatrics, Division of Immunology, Allergy, and Retrovirology, Baylor College of Medicine and Texas Children′s Hospital, Houston and The Woodlands, USA; 3 Biochemistry and Molecular Biology, Institute of Theoretical Medicine, Faculty of Medicine, University of Augsburg, Augsburg, Germany; 4 Center for Interdisciplinary Health Research, University of Augsburg, Augsburg, Germany; 5 Center for Advanced Analytics and Predictive Sciences (CAAPS), University of Augsburg, Augsburg, Germany; 6 Institute of Immunology, Faculty of Medicine Carl Gustav Carus, Technische Universität Dresden, Dresden, Germany; 7 National Center for Tumor Diseases (NCT), Partner Site Dresden, Dresden, Germany; 8 German Cancer Consortium (DKTK), Partner Site Dresden, Dresden, and German Cancer Research Center (DKFZ), Heidelberg, Germany; 9 Institute of Clinical Chemistry and Clinical Pharmacology, University Hospital Bonn, Bonn, Germany

**Keywords:** Intramembrane proteolysis, Signal peptide peptidase, Aicardi-Goutières syndrome, ER-associated protein degradation, cGAS/STING pathway, Cytosolic DNA degradation

## Abstract

Signal peptide peptidase (SPP) is an ER-resident aspartyl intramembrane protease cleaving proteins within type II-oriented transmembrane segments. Here, we identified the tail-anchored protein Three prime repair exonuclease 1 (TREX1) as a novel substrate of SPP. Based on its DNase activity, TREX1 removes cytosolic DNA acting as a negative regulator of the DNA-sensing cGAS/STING pathway. TREX1 loss-of-function variants cause Aicardi-Goutières syndrome (AGS), a type I interferonopathy. Cleavage of ER-bound TREX1 by SPP releases a cleavage product into the cytosol. Proteolysis depends on sequence determinants within the transmembrane segment and is modulated by different disease-associated TREX1 variants. The AGS-causing T303P variant greatly enhanced susceptibility of TREX1 to intramembrane cleavage accounting for increased degradation and reduced protein stability in AGS patients homozygous for this variant. Other variants within the TREX1 transmembrane segment, P290L, Y305C and G306A, associated with systemic lupus erythematosus variably modulated TREX1 proteolytic processing. Altogether, intramembrane proteolysis can act as a regulator of TREX1 both by controlling its cytosolic localization and mediating its turnover with implications for disease pathogenesis.

**Supplementary Information:**

The online version contains supplementary material available at 10.1007/s00018-025-05645-5.

## Introduction

Intramembrane proteolysis describes the cleavage of substrate proteins within transmembrane segments by a membrane-embedded protease as it was initially discovered for the Sterol regulatory element binding protein (SREBP) and the Amyloid precursor protein (APP) [1]. By this means, cleavage fragments are liberated to either side of the membrane. Signal peptide peptidase (SPP) is an aspartyl intramembrane protease with homology to the presenilins, the catalytic subunits of the γ-secretase complex [2, 3]. SPP was initially identified based on its ability to process selected signal peptides within the membrane of the endoplasmic reticulum (ER) after these have been released from newly synthesized proteins [4]. Furthermore, SPP can process certain viral proteins in virus-infected cells required for replication of the respective viruses [2, 5]. Among physiological SPP substrates, ER-resident tail-anchored (TA) proteins have come into the focus over recent years [6–10]. TA proteins exhibit a single C-terminal transmembrane segment in type II orientation (N-terminus facing the cytosol) and are inserted into cellular membranes post-translationally [11]. With their membrane topology and short luminal domain they fulfill the general substrate requirements of SPP [12]. Cleavage of TA proteins by SPP often initiates their degradation with cleavage fragments rapidly being handed over to other proteolytic pathways like the proteasome [13]. However, stable cleavage fragments of certain TA proteins like heme oxygenase 1 (HO-1) are released into the cytosol and have biological functions [7]. Mechanisms determining the fate of SPP-generated cleavage fragments are currently poorly understood.

Three prime repair exonuclease 1 (TREX1) [14] is a DNase with 3′−5’ exonuclease activity [15, 16] and rather ubiquitous expression [17]. It can release single nucleoside monophosphates from the 3′ end of both double- and single-stranded DNA [15]. *TREX1* pathogenic variants in humans were found associated with autoimmune and inflammatory diseases [18, 19]. These include Aicardi-Goutières Syndrome (AGS), Familial Chilblain Lupus (FCL) or Retinal Vasculopathy with Cerebral Leukodystrophy (RVCL) [14]. AGS is a severe neuroinflammatory encephalopathy associated with other systemic manifestations and a high early mortality or significant disability [20]. AGS patients exhibit an excessive production of type I interferons causing immune cell activation and inflammatory organ damage [20]. Consistent with the central task of TREX1 to degrade DNA reaching the cytosol, loss of TREX1 function results in cytosolic DNA accumulation [14, 16] triggering the DNA-sensing cGAS-STING pathway [21]. Via activation of the transcription factor IRF3, this leads to the uncontrolled production of type I interferons seen in AGS patients. This model is supported by *Trex1* knockout mice, which also display inflammatory phenotypes [22, 23].

TREX1 is considered to be a TA protein which is anchored to the membrane of the endoplasmic reticulum via a C-terminal transmembrane segment [14, 17, 24] so that the N-terminal DNase domain faces the cytosol [25]. Being an integral membrane protein, presence of TREX1 in the nucleus as described by several studies remains mechanistically unexplained [26–30]. Interestingly, different disease-associated TREX1 variants affecting the transmembrane segment have been reported [19, 20]. Biallelic TREX1 T303P variant causes AGS [20], whereas three monoallelic missense variants (P290L, Y305C, G306A) seem to increase the risk for systemic lupus erythematosus (SLE) [19]. Since the transmembrane segment is far apart from the catalytic site, it is currently unclear how these mutations may affect TREX1 function and cause disease.

As an ER-resident TA protein, we considered TREX1 as a substrate candidate for SPP. We report that SPP cleaves TREX1 liberating the cytosolic domain from the membrane. This intramembrane cleavage fulfills a dual function by also mediating turnover of TREX1 and regulating cellular TREX1 levels. Finally, we demonstrate that proteolytic processing of TREX1 is modulated by different disease-associated variants.

## Material and methods

### Cell lines

HeLa, HEK293T (HEK) and THP-1 cells were obtained from DSMZ. Generation of SPP-deficient HEK cells by CRISPR/Cas9 has been described before [31]. Cells were kindly provided by Marius Lemberg, University of Cologne. TREX1- deficient HeLa cells have been reported in [32]. Cell lines with stable, doxycyclin-inducible overexpression of catalytically active (SPP wild type) or inactive SPP (SPP D/A) in the T-REx™−293 Cell Line (Invitrogen) have been described before [33].

### AGS patient cells

Informed consent for research was obtained from the parent of the subjects under protocol H-21453 approved by the Baylor College of Medicine Institutional Review Board. All studies were performed in accordance with the guidelines established by the Declaration of Helsinki. Blood samples were collected for peripheral blood mononuclear cell (PBMC) isolation. Skin biopsies were obtained from each research subject and placed aseptically in RPMI 1640 medium. The specimens were then shipped to a commercial company (Baylor Genetics, USA) to generate dermal fibroblasts. Control human dermal adult fibroblasts were purchased from either ATCC (PCS-201-012) or Thermo Fisher Scientific (C0135C). To validate the *TREX1* genotype of the cell lines, cells were lysed in Direct PCR tail lysis buffer (VWR) supplemented with 200 µg/ml proteinase K (Roth) at 56 °C overnight. Following an inactivation step at 85 °C for 45 min, a sequence encompassing the entire human *TREX1* ORF was amplified from the genomic DNA by PCR employing the Phusion Polymerase (Thermo Fisher Scientific) and the following primers: hTREX1-PCR-Fw: GAAGGCCTGAGATGTGCTTCTGC, hTREX1-PCR Rv GAGGTCTGTGCTGCCAAGAAGGG. After separation on 1.5% agarose gels equipped with SYBR Safe DNA Gel Stain (Thermo Fisher Scientific), the PCR fragments were isolated using the High Pure PCR Product Purification Kit (Roche) according to the manufacturer’s instructions. The complete *TREX1* coding sequence was subsequently sequenced with the help of Mycrosynth Seqlab using the following primers: hTREX1-Seq1-Fw GATCTTAACACTGGGCACTCAC, hTREX1-Seq2-Fw GCCCAAGGAAGAGCTATAGC.

### Plasmids

The human TREX1 open reading frame (AF319569.1) was fused to an N-terminal HA epitope by PCR using HA-hTrex-1-BamHI-fw (ATCGGATCCGGCACCATGTACCCATACGACGTCCCAGACTACGCTGGCTCGCAGGCCCTGCC) and HA-hTrex-1-XhoI-rev (TAGTCTCGAGCTACTCCCCAGGTGTGGCCAGGGATAGTCCATACAGTGTGGCTAC) as forward and reverse primers and inserted into the pcDNA3.1/Hygro + backbone (Invitrogen) utilising BamHI and XhoI. By using longer reverse primers, which cover the site of the intended mutation, P290L, Y305C, G306A and T303P mutant constructs of human Trex1 were generated by PCR. The respective inserts were inserted into the pcDNA3.1/Hygro + vector with the same pair of enzymes. To create expression constructs with a C-terminal HA epitope and an unaltered N-terminus, the inserts from the existing wild type and mutant TREX1 constructs were amplified by PCR using hTrex1-HA HindIII fw (ATATAAGCTTGCCACCATGGGCTCGCAGGCCCTGCC) and hTrex1-HA XhoI rev (ATATCTCGAGCTAAGCGTAATCTGGAACATCGTATGGGTACTCCCCAGGTGTGGCCAGGG) as flanking primers so that the sufficiently short reverse primer did not cover any of the previously introduced mutation sites. The C-terminally HA tagged TREX1 constructs were inserted into the same backbone by the combination of HindIII and XhoI. The open reading frame of TREX1-ΔCT mutant (aa 1–286) devoid of the 28 C-terminal amino acids of the wild type protein was amplified using hTrex1 ΔCT rev (ATTCTCGAGCTACCCCTCCCTGGATAGGGCTCC) as reverse primer. The PCR product was cloned as described above using HindIII and XhoI. In order to generate a non-cleavable TREX1 mutant, the transmembrane domain (TMD) of TREX1 (aa 288–309) was exchanged by the corresponding stretch of human HO2 (aa 296–315) by two consecutive overlap extension PCRs employing hTrex1 HO2 rev 1 (CCAAGAGTCCAGCAGCTAGGGCCACACCAGCGGCCAGGATGAACTGGAGCAGCCCCTCCCTGGATAGG) and hTrex1 HO2 rev 2 XhoI (TAGTCTCGAGCTACTCCCCAGGTGTGGCGTAGTACCAGGCCAAGAGTCCAGCAGCTAGGGCCACACCAGCGGCCAGGATG) reverse primers. To generate a C-terminally HA tagged and N-terminally unaltered HO2 chimeric construct, the previously generated construct was amplified in a PCR reaction using a new set of flanking primers hTrex1 HO2-TMD HA fw (ATATAAGCTTGCCACCATGGGCTCGCAGGCCCTGCC) and hTrex1 HO2-TMD rv (ATATCTCGAGCTAAGCGTAATCTGGAACATCGTATGGGTACTCCCCAGGTGTGGCGTA). The C-terminally HA tagged HO2-TMD chimeric construct was inserted into pcDNA3.1 backbone via restriction and ligation with the HindIII and XhoI enzyme pair. The orf encoding human SPP (NM_030789.3) equipped with a C-terminal myc-tag was amplified using the following primers: hSPP-HindIII-fw (ATAAGCTTGCCACCATGGACTCGGCCCTCAGCGATCCGCATAACGG), hSPP-myc-XhoI-rev (AGTCTCGAGTCACAGATCCTCTTCTGAGATGAGTTTTTGTTCTTTCTCTTTCTTCTCCAGCC). The inactivating D280A mutation was integrated by site directed mutagenesis employing the following oligonucleotides: hSPP-D280A-fw (CATTGCCTTGCTGCTGCGCTTTGCCATCAGCTTGAAGAAGAATACCC), hSPP-D280A-rev (GGGTATTCTTCTTCAAGCTGATGTCAAAGCGCAGCAGCAAGGCAATG). The coding sequences were inserted into pcDNA3.1 hygro ( +) using the attached HindIII and XhoI sites. All generated plasmids were finally controlled by Sanger sequencing with the help of Microsynth Seqlab and Azenta.

### Cell culture and transfection

HeLa, HEK293T, dermal fibroblasts and the T-REx™−293-based cell lines were cultured in DMEM (Thermo Fisher Scientific) supplemented with 10% Fetal Bovine Serum (Thermo Fisher Scientific), 100 U/ml penicillin and 100 µg/ml streptomycin (both from Thermo Fisher Scientific). THP-1 cells were maintained in RPMI (Thermo Fisher Scientific) with the same supplements. The T-REx™−293-derived cell lines were cultivated with the addition of 10 μg/ml of the selection antibiotic blasticidin (InvivoGen). Adherent cells were detached with Accutase (Thermo Fisher Scientific) for passaging. All cell lines were kept at 37 °C in a humidified atmosphere with 5% CO₂. Cells were seeded one day before experiments or transfections. Transfection with expression plasmids was achieved with polyethylenimine (PEI, Polyscience) with a μg DNA/ μg PEI ratio of 1:2.5. To reduce toxicity, the medium of transfected cells was replaced 4–6 h after the transfection. Inhibitor X (Biotechne/Tocris) was dissolved in DMSO and applied at final concentrations of 1 or 2 µM in the cell culture medium as specified in the legends. Bortezomib (Tocris bioscience), dissolved in DMSO, was applied in a final concentration of 20 nM. For chase experiments, cycloheximide (Sigma), dissolved in cell culture grade water, (Sigma) was employed at 100 µg/ml. To interfere with lysosomal acidification and proteolysis, ammonium chloride (Roth, dissolved in water) was added to the culture medium at a concentration of 25 mM. In case of transiently transfected cells, the compounds were applied at the time of the post-transfection medium replacement. Control samples were treated with the corresponding amount of the respective solvent.

### Cell lysis and Western Blotting

Protein extraction from the cells was conducted as previously described [34] with a lysis buffer containing 50 mM Tris/HCl, pH 7.4, 150 mM NaCl, 1% (w/v) Triton X-100 (Sigma) and 0.1% SDS (Roth) supplemented with 1X cOmplete (Roche), 4 mM Pefabloc (Serva), 1 μg/mL Pepstatin A (Roth) and 4 mM EDTA (Roth) for inhibition of proteases. The protein concentration of individual lysates was determined by utilizing the Pierce™ BCA Protein Assay Kit following the manufacturer’s instructions. Following SDS-PAGE separation of proteins with a standard tris–glycine buffer system, semi-dry transfer onto nitrocellulose membranes was achieved as previously described [34]. For immunodetection, the following primary antibodies were utilized: anti-human-TREX1 (D8E20, Cell Signaling); anti-HA (3F10, Roche); anti-Tubulin (2144S, Cell Signaling); anti-Calnexin (C4731, Sigma); anti-GAPDH (W17079A, BioLegend); anti-GFP (2956, Cell Signaling). For detection of human and murine SPP, a custom-made polyclonal antiserum was generated against the synthetic peptide AETESKEESTEASASKRLEKKEK and affinity-purified against the immunogen by Pineda Antikörper-Service. Horseradish peroxidase-coupled secondary antibodies were obtained from Dianova. Chemoluminescent detection was performed with a solution of 250 μg/mL Luminol (Roth), 110 μg/mL p-Coumaric acid (Sigma), 1/24 Lumigen A (Bioquote), 1/24 Lumigen B (Bioquote) and 1/400 30% H_2_O_2_ (Roth) and images were captured using an Amersham Imager 600 (GE Healthcare). Images were processed with GNU Image Manipulation Program Software (GIMP, version 2.10.32) and analysed densitometrically using Image J (version 1.52a). Determined band intensities were divided by those of the proteins detected as loading control. When applicable, the data was normalized to the control sample as indicated in the respective figures. When transiently transfected HEK and SPP-deficient HEK cells were compared for TREX1 steady state levels, the data was further normalized to that of co-expressed GFP to minimize the effect of potentially differing transfection efficiencies between the cell lines.

### Subcellular fractionation

Separation of cytosolic and ER-associated TREX1 was performed according to a procedure reported in [35] with some modifications described in the following. Cells were collected via scraping in phosphate buffered saline (PBS) supplemented with 1X cOmplete (Roche) and recovered by centrifugation at 210x*g*. The pellet was resuspended in ice cold hypotonic buffer containing 10 mM HEPES–NaOH, pH 7.4, adjusted with 1X cOmplete (Roche), 4 mM Pefabloc (Serva) as well as 5 μg/ml pepstatin A (Roth) and incubated on ice for 10 min. Subsequently, cells were mechanically disrupted by 15 passages through a 30G cannula using a 1 ml insulin syringe. The sample was mixed with an equal volume of 2X Gradient Buffer (90 mM HEPES–NaOH, pH 7.4, 300 mM NaCl, 50 μg/mL digitonin, protease inhibitors as above) and the homogenization procedure was repeated for another 15 strokes. After an incubation on ice for 10 min, debris and nuclei were removed from the homogenate by centrifugation for 5 min at 750x*g*. The post-nuclear supernatant (1.2 ml) was loaded into 14 ml ultra-clear centrifuge tubes (Beckman), which was then under-layered with a 11.4 ml 10%–60% (w/v) sucrose gradient and centrifuged at 40,000 rpm (285,000x*g*_*max*_) at 4 °C for 2 h in a SW 40Ti rotor (Beckman). Twelve fractions of 1 ml and a final fraction of 600 μl were collected from top to bottom and analysed by Western blotting.

### Indirect immunofluorescence

1 × 10^5^ Hela or ΔTREX HeLa cells were seeded on glass cover slips in 6-well plates. On the following day, cells were transfected as described above. One day post-transfection, the cells were fixed with 4% (w/v) PFA and permeabilized with 0.2% (w/v) saponine, both in PBS, as described before [34]. Primary antibodies used in this procedure include anti-TREX1 (D8E20, Cell Signalling), anti-HA (3F10, Roche), anti-Myc (2278S, 2276S, Cell signalling) and anti-Calnexin (C4731, Sigma). Visualization was achieved with Alexa-488 or Alexa-594 coupled secondary antibodies by Life Technologies. The slides were mounted in Mowiol (Merck) supplemented with DABCO [1,4-diazobicyclo-(2.2.2) octane] (Sigma) and DAPI (4-,6-diamidino-2-phenylindole, Sigma) for the visual detection of the nucleus. Pictures of the slides were acquired with a Leica Stellaris 8 Confocal Microscope using 63 × oil immersion objective and the LAS X software. Further processing of the images was done with ImageJ.

### Quantitative real-time PCR

RNA was isolated from peripheral blood mononuclear cells (PBMC) or fibroblasts employing the NucleoSpin RNA Plus Mini kit (Macherey–Nagel) according to the manufacturer’s instructions. Following RNA isolation, identical amounts of RNA were transcribed into cDNA employing the RevertAid™ First Strand cDNA Synthesis Kit (Thermo Fisher Scientific) in a total volume of 20 µl. 0.5 µl of cDNA were subsequently analysed by quantitative real-time PCR employing the Maxima™ SYBR™ Green/ROX 2 × qPCR Master Mix (Thermo Fisher Scientific). The PCR was run using a CFX384 TouchTM Real-Time PCR Detection System (Bio-Rad). The following primers were employed: hTREX1 fw: GTGTGCAGCCGAGTCACTAC; hTREX1 rev: AGTGGCCTCCATGTCGAAAA; hTUBA1A fw: GTCGCGCTGTAAGAAGCAAC; hTUBA1A rev: GCATTGCCAATCTGGACACC; hGAPDH fw: GTGAAGGTCGGAGTCAACGG; hGAPDH rev: TGACAAGCTTCCCGTTCTCA; hTBP fw: CGCCGGCTGTTTAACTTCG; hTBP rev: TGGGTTATCTTCACACGCCA; hHPRT fw: GACCAGTCAACAGGGGACAT; hHPRT rev: AAGCTTGCGACCTTGACCAT. Ct values of *TREX1* as determined by the Bio-Rad CFX Maestro software (version 2.2) were normalized to the mean Ct value of the four housekeeping genes *GAPDH*, *TUBA1A*, *HPRT* and *TBP*. Relative expression of TREX1 was finally calculated as described in [36].

### PBMC isolation

Whole blood was collected from the two AGS patients, their mother and a non-related healthy volunteer in heparinized tubes. After addition of 20 ml of phosphate buffered saline (PBS), the resulting 35 ml were carefully layered beneath 15 ml of Ficoll-Paque within a 50 ml conical tube. Samples were centrifuged at 400x*g* for 20 min at room temperature with no brake. The PBMC layer at the interface between plasma and Ficoll was carefully collected using a sterile pipette and transferred to a new 50 mL conical tube. PBMCs were washed with 20 mL of PBS and centrifuged at 300x*g* for 10 min. The supernatant was discarded, and the cell pellet was resuspended in RPMI 1640 medium containing 10% fetal bovine serum.

For the analysis of endogenous TREX1 in primary human immune cells, PBMC were isolated from buffy coats of healthy donors generated by the German Red Cross Blood Donation Service North-East, Dresden. Donors gave their written informed consent and the study was approved by the local institutional review board of the Faculty of Medicine Carl Gustav Carus, TU Dresden, Germany (EK138042014). Blood was diluted 1:1 with PBS, layered on Ficoll-Hypaque solution (Biochrom) and density centrifugation was performed for 20 min, 980 × *g* at room temperature. The interphase was harvested and PBMC were washed twice with cold PBS.

### Statistical analysis

Calculations were performed with Microsoft Excel 2016, whereas results were statistically analysed with the GraphPad Prism software version 9.4.1. Diagrams show mean values ± SD, with the statistical test employed indicated in respective figure legends. N represents the number of independent experiments, while n indicates the number of independent samples. Figures and schemes were assembled using Adobe Illustrator software.

## Results

### TREX1 is a substrate of the intramembrane protease SPP

**Fig. 1:**
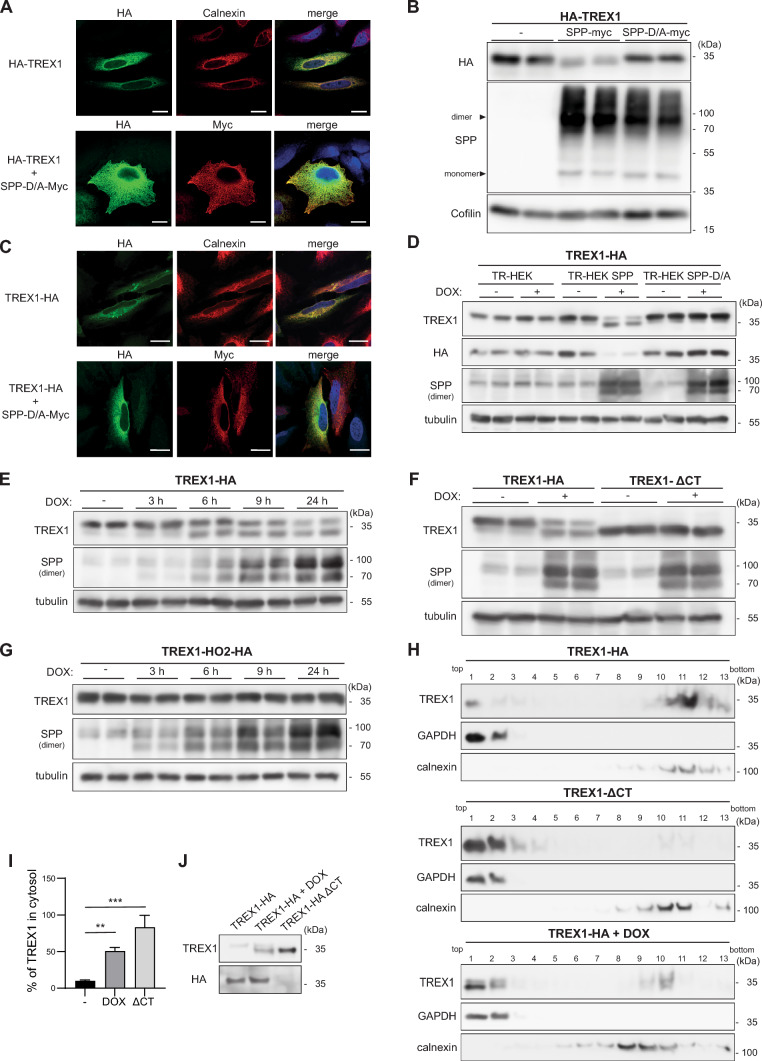
TREX1 is a substrate of the intramembrane protease SPP. **A** HeLa cells were transiently transfected with either N-terminally HA-tagged TREX1 (HA-TREX1) alone or together with proteolytically inactive SPP (SPP-D/A) with a C-terminally fused Myc epitope. After fixation, cells were analysed by indirect immunofluorescence employing the indicated antibodies. Calnexin served as ER marker protein. Scale bar, 20 µm. **B** HEK cells were transfected with HA-TREX1 and either wild type or inactive (D/A) SPP-myc. Processing of TREX1 was subsequently monitored by Western Blotting. **C** The experiment described in **A** was repeated with C-terminally HA-tagged TREX1 (TREX1-HA). Scale bar, 10 µm. **D** T-REx HEK cells inducibly expressing SPP (TR-HEK SPP) or an inactive variant (TR-HEK SPP-D/A) or non-transfected control cells (TR-HEK) transiently overexpressing TREX1-HA were treated for 24 h with 10 µg/ml doxycycline (DOX) as indicated. Upon cell lysis, TREX1 protein levels were evaluated by Western Blotting. **E** TR-HEK SPP cells were transiently transfected with TREX1-HA and treated with 10 µg/ml doxycycline for the indicated periods prior to Western Blot analysis of TREX1 processing by SPP. **F** Following transfection with either wild type TREX1-HA or a mutant lacking the C-terminal transmembrane domain (TMD) (TREX1-ΔCT), TR-HEK SPP cells were treated with 10 µg/ml doxycycline as indicated for 24 h. Cell lysates were finally analysed by Western Blotting. **G** The experiment described in **E** was repeated with a mutated version of TREX-1 HA in which its TMD was exchanged by that of HO2, which is not a substrate of SPP. **H** Following transfection with either wild type TREX1-HA or a variant lacking the C-terminal transmembrane domain (TMD) (TREX1-ΔCT), TR-HEK SPP cells were treated with 10 µg/ml doxycycline as indicated (+ DOX) for 24 h. Subsequently, the cells were subjected to subcellular fractionation. The distribution of TREX1 in the gradient fractions was analysed by Western Blotting. GAPDH and calnexin were detected as marker proteins of the cytosol and ER membranes, respectively.** I** Cytosolic TREX1 in relation to the total TREX1 amounts detected over all gradient fractions was quantified from blots as depicted in H). N:3, n:3. One-Way ANOVA with Dunnett’s post hoc test. ** p ≤ 0.01; *** p ≤ 0.001. **J** Fraction #1 from gradients as described in **H** were loaded next to each other to directly compare the molecular weight of the different forms of TREX1 from the different samples. From the TREX1-ΔCT fractionation, only one third of the amount in relation to the other samples was loaded to achieve comparable band intensities for size comparison

TREX1 is an ER-resident TA protein and was shown to colocalize with SPP in HeLa cells (Fig. 1A), so that it fulfils the basic requirements of being a substrate of this intramembrane protease. Therefore, we tested this in a protease-substrate co-expression assay in HEK cells (Fig. 1B). Total TREX1 levels were reduced by wild type SPP, but not by the catalytically inactive SPP D265A mutant, which was indicative of cleavage. SPP was primarily detected as a dimer, why in the following only bands corresponding to the dimeric form are shown. Due to the short C-terminus of TREX1 the potential proteolytic fragment could not be distinguished from the uncleaved precursor. Therefore, we generated a TREX1 construct with a C-terminal HA tag to increase the molecular weight difference between these two forms. The TREX1-HA fusion protein was targeted to the ER (Fig. 1C) indistinguishable from the previously employed N-terminally tagged variant (Fig. 1A). We expressed TREX1-HA in cell lines with doxycycline-inducible expression of catalytically active (TR-HEK SPP) or inactive SPP (TR-HEK SPP-D/A) (Fig. 1D). A single band corresponding to TREX1 was visualized upon detection via its C-terminal HA tag. The intensity of this band was reduced, when expression of catalytically active SPP was induced. However, when detection with a commercial antibody directed against the cytosolic domain was performed an additional band of slightly lower molecular weight upon induction of SPP overexpression was observed. In control cells or upon expression of catalytically inactive SPP, no effects on the TREX1 band pattern were seen. Altogether, these observations are consistent with an intramembrane cleavage releasing the short C-terminus from the TREX1 protein, though this cleavage fragment with its predicted size of ~ 20–30 amino acids could not be detected by the applied electrophoretic system. We performed a time course of SPP induction (Fig. 1E) which demonstrated a gradual transition of the TREX1 precursor to the N-terminal cleavage product, which in this system exhibited a relevant stability. The apparent molecular weight of the observed cleavage product corresponded well to a TREX1 ΔCT variant (aa 1–286) comprising the entire cytosolic domain of TREX1 (Fig. 1F). In absence of the transmembrane segment, TREX1 ΔCT was not cleaved by the intramembrane protease SPP. Though the substrate determinants recognized by SPP are not fully defined, it is accepted that features within the substrate’s transmembrane domain are critical. We probed this concept by exchanging the TREX1 transmembrane domain by that of heme oxygenase 2 (HO2) (Suppl. Figure 1), a proven non-substrate of SPP [37]. When expressed in the TR-HEK SPP cell line, no proteolysis of this chimeric protein was detected upon SPP induction (Fig. 1G) confirming that the TREX1 TMD contains critical determinants for cleavability. Next, we asked whether the intramembrane cleavage by SPP is able to release the TREX1 cleavage fragment into the cytosol or whether this remains associated with the ER membrane. Therefore, we employed sucrose density gradient centrifugation to separate cytosol and ER membranes. As depicted in Fig. 1H, the assay was suitable to discriminate between these subcellular compartments demonstrated by Western Blot analysis of the marker proteins GAPDH (cytosol) and calnexin (ER). Wild type TREX1 was predominantly found in the ER-containing fractions in line with the immunofluorescence results (Fig. 1C). As expected, TREX1 ΔCT was not detected in the ER fraction due to lack of a membrane-anchor and remained in the cytosol. Upon SPP overexpression also wild type TREX1 largely shifted to the cytosol-containing fractions of the gradient as quantified in Fig. 1I. Surprisingly, also small amounts of the uncleaved precursor could be reproducibly detected within the gradient top fractions under the employed overexpression conditions (Fig. 1J). This finding might relate to the presence of non-integrated full length TREX1 which is inserted into ER membranes post-translationally. Overall, our data confirm that TREX1 is released into the cytosol by SPP cleavage.

### Soluble, SPP-cleaved TREX1 is present endogenously

**Fig. 2:**
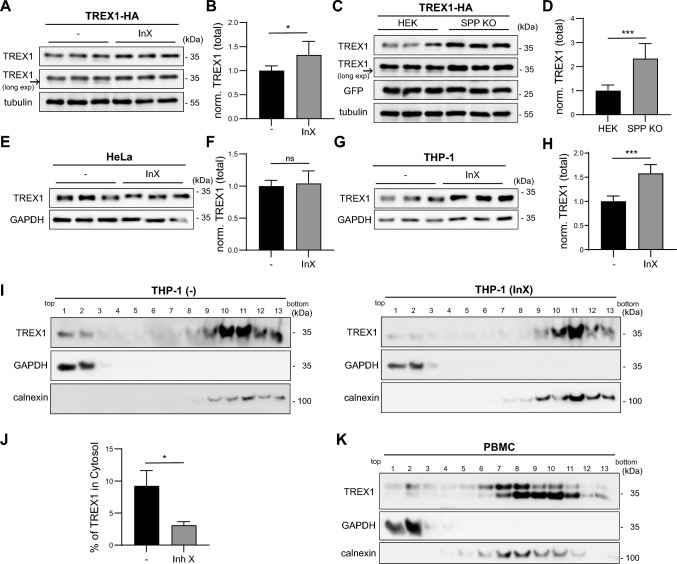
SPP releases endogenous TREX1 into the cytosol.** A** Following transient transfection with C-terminally HA-tagged TREX1 (TREX1-HA), HEK cells were either treated with 2 μM inhibitor X (L 685,458, InX) or DMSO as control for 24 h. Processing of TREX1 was subsequently monitored by Western Blotting. **B** Densitometric quantification of total TREX1 levels normalized to tubulin in transiently transfected TREX1-HA with or without inhibitor X treatment as shown in **A**. N:2, n:6. Unpaired two-tailed Student’s t-test. **C** Wild type and SPP-deficient (SPP KO) HEK cells were transiently transfected with TREX1-HA and GFP. Subsequently, TREX1 and GFP levels were monitored by Western Blotting. **D** Densitometric quantification of total TREX1 levels normalized to co-transfected GFP and tubulin upon transient expression of TREX1-HA in wild type and SPP deficient (SPP KO) HEK cells as shown in **C**. N:3, n:9. Unpaired two-tailed Student’s t-test. **E** HeLa cells were treated either with 1 μM inhibitor X or DMSO as control for 24 h. Endogenous TREX1 levels were monitored by Western Blotting. **F** Quantification of normalized TREX1 band intensities in relation to DMSO-treated controls. N:3, n:9. Unpaired two-tailed Student’s t-test. **G** THP-1 cells were treated either with 1 μM inhibitor X or DMSO as control for 24 h. Endogenous TREX1 levels were analysed by Western Blotting. **H** Quantification of the inhibitor X effect on total TREX1 levels normalized to GAPDH in THP-1 cells **F**. N:2, n:6. Unpaired two-tailed Student’s t-test. **I** THP-1 cells were treated with 1 μM inhibitor X or DMSO as control for 48 h. After subcellular fractionation, TREX1 levels in the gradient fractions were determined by Western Blotting. **J** Quantification of the cytosolic fraction of total cellular TREX1 in THP-1 after subcellular fractionation of the representative experiment depicted in **I**. Cytosolic TREX1 is expressed as a percentage of the total TREX1 detected over the entire gradient. N:1, n:3. Unpaired two-tailed Student’s t-test. **K** The distribution of endogenous TREX1 in ER and cytosolic fractions of human PBMCs was analyzed by subcellular fractionation and subsequent Western Blotting. ns, not significant; *, p ≤ 0.05; ***, p ≤ 0.001

We aimed to analyze whether cleavage of TREX1 also occurs under endogenous conditions. Upon transfection of TREX1-HA into HEK cells, a very faint band below the TREX1 precursor was detected, which could represent the cleavage fragment (Fig. 2A, arrow). Importantly, this band was diminished by pharmacological inhibition of SPP with the aspartyl intramembrane protease inhibitor X (Fig. 2A), which was associated with a significant increase of the overall TREX1 abundance (Fig. 2B) [38]. This is consistent with overexpressed TREX1 being cleaved by endogenous SPP, though steady-state levels of the cleavage fragment were rather low. We repeated this analysis in SPP-deficient HEK cells [31] (Suppl. Fig. 2, Fig. 2C, D) showing a depletion of the presumed cleavage fragment (arrow). Similar to pharmacological inhibition, also loss of SPP resulted in an increase of total cellular TREX1 levels (Fig. 2D) suggesting that SPP-mediated cleavage of TREX1 facilitates the turnover of the protein. Since HEK cells do not express TREX1, we switched to HeLa cells (Fig. 2E, F) to analyse TREX1 cleavage at full endogenous level. Here, SPP inhibition resulted in a shift of TREX1 towards a slightly increased molecular weight, while overall TREX1 abundance was not affected. The band shift was also seen in the monocytic THP-1 cell line upon treatment with inhibitor X (Fig. 2G). In addition, SPP inhibition also led to a significant stabilization of TREX1 levels in these cells (Fig. 2H) reminiscent of the experimental results in HEK cells (Fig. 2A–D). Using subcellular fractionation, we detected endogenous TREX1 in the cytosolic fractions of THP-1 cells (Fig. 2I, J). The cytosolically localized TREX1 was partially re-distributed to the membrane fraction by adding the SPP inhibitor X as demonstrated by a lower TREX1 cytosol/membrane ratio. Since all experiments so far were cell line-based, we wanted to confirm if soluble TREX1 is also present in primary cells. Therefore, we performed density gradient separation on human PBMCs (Fig. 2K). Endogenous TREX1 was detected as a double band. Similar to the situation in THP-1 cells, a small part of the endogenous TREX1, in particular of the upper band, was present in the cytosol. Altogether, we conclude that intramembrane cleavage of endogenous TREX1 by SPP occurs similarly to overexpressed TREX1 in cell lines fulfilling a dual function by generating soluble cytosolic TREX1 and contributing to TREX1 degradation.

### SPP and the proteasome system independently contribute to TREX1 turnover

**Fig. 3:**
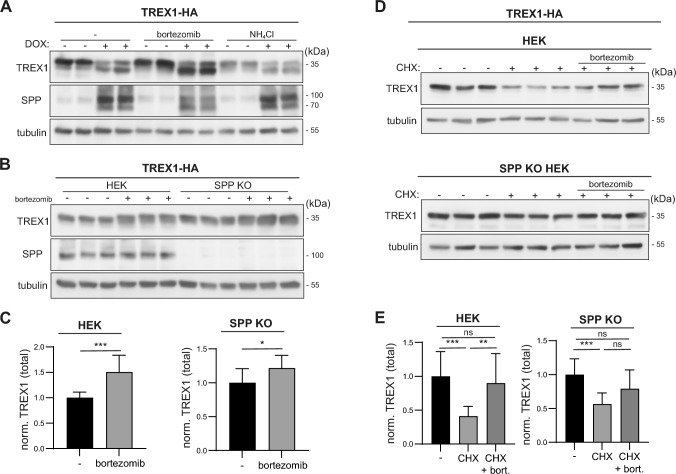
SPP and the proteasome system contribute to turnover of TREX1. **A** TR-HEK SPP cells were transiently transfected with TREX1-HA. Subsequently, SPP expression was induced with 10 µg/ml doxycycline (DOX) for 24 h as indicated. Additionally, some cells were incubated for the same time with either 20 nM bortezomib or 25 mM ammonium chloride to inhibit the proteasome or lysosomal degradation, respectively. TREX1 levels were subsequently evaluated by Western Blotting. **B** Wild type or SPP-deficient (SPP KO) HEK cells were transfected with TREX1-HA and subsequently treated for 24 h with 20 nM bortezomib. TREX1 protein levels were visualized by Western Blotting. **C** The amount of TREX1 was quantified densitometrically from blots as depicted in **B** and normalized to tubulin. Data were normalized to the mean of the DMSO-treated controls for both cell lines individually. N:3, n:9. Unpaired two-tailed Student’s t-test. **D** Wild type as well as SPP KO HEK cells transiently expressing TREX1-HA were treated for 9 h with 100 µg/ml cycloheximide (CHX) to block protein synthesis. Where indicated, cells were additionally treated with 20 nM bortezomib during this period. Processing of TREX1 was monitored by Western Blotting. **E** TREX1 levels were quantified densitometrically and normalized to those of the corresponding loading controls as depicted in **D**. The amount of TREX1 was normalized to that of the DMSO-treated controls. N:4, n:12. One-Way ANOVA with Tukey’s post hoc testing. ns, not significant; *, p ≤ 0.05; ** p ≤ 0.01; *** p ≤ 0.001

Cleavage products generated by members of the SPP/SPPL are often rapidly degraded. In most cases, this is facilitated by the ubiquitin–proteasome system. To test whether also the TREX1 soluble domain released by SPP is degraded by this pathway, we treated TR-HEK SPP cells expressing TREX1-HA with either bortezomib, an established proteasome inhibitor, or ammonium chloride to interfere with lysosomal degradation. As depicted in Fig. 3A, bortezomib clearly stabilized soluble TREX1 generated by SPP cleavage. In parallel, also a slight stabilization of the full length precursor was observed. By contrast, ammonium chloride did not have a clear impact on the amounts of any of the two TREX1 species. While clearly documenting a role of the ubiquitin–proteasome system for the turnover of soluble TREX1, these findings also suggest a potentially SPP-independent impact on membrane-embedded full length TREX1. We aimed to corroborate this by employing our SPP-deficient HEK cell line. As demonstrated before in Fig. 2A, these cells accumulated more TREX1-HA upon transfection with the corresponding construct than the wild type control cell line (Fig. 3B, C). In both cell lines, treatment with bortezomib for 24 h significantly increased TREX1-HA levels, even though slightly more pronounced in the wild type cells, indicating a significant contribution of the ubiquitin–proteasome system to the turnover of membrane-bound TREX1. To estimate the contribution of both degradative pathways, we blocked protein synthesis of HEK cells transiently expressing TREX1-HA with cycloheximide alone or in combination with bortezomib (Fig. 3D, E). In the 9 h chase period, TREX1-HA levels decreased by approximately 60% in wild type cells, which was significantly less pronounced in the presence of bortezomib. In HEK cells lacking endogenous SPP expression, the picture was overall similar, however the cycloheximide-dependent decrease in TREX1-HA was lower than in the corresponding wild type cells reflecting the contribution of SPP to TREX1 degradation. Given the strong effect of cycloheximide treatment on TREX1 levels also in SPP-deficient cells, these findings point towards the activity of at least two degradative pathways controlling the stability of full length TREX1, among which SPP-mediated intramembrane proteolysis exclusively facilitates the liberation of the TREX1 ICD.

### SLE-associated TREX1 TMD variants differentially affect cleavage by SPP

**Fig. 4:**
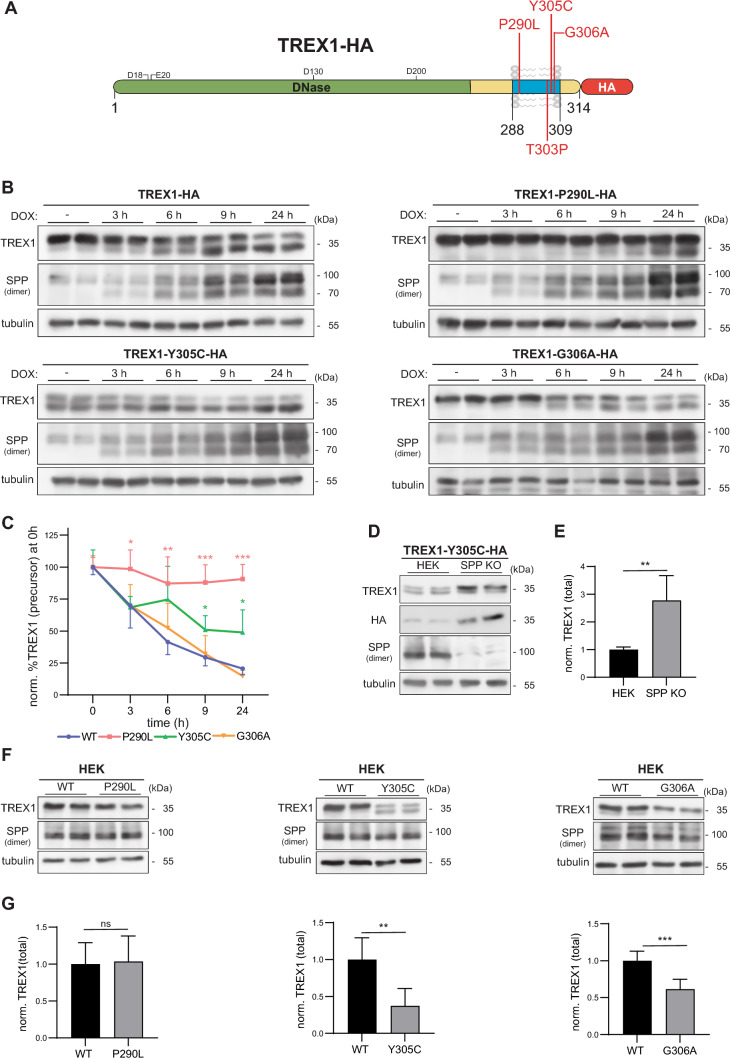
The impact of SLE-associated TREX1 variants on cleavage by SPP. **A** Graphical representation of TREX1 with its domains and the locations of SLE-associated point mutations and the AGS-causing T303P point mutation. Green: part of the protein containing the DNAse catalytic domain. According to structural data, the indicated amino acid residues in this section contribute to binding of DNA substrates [25]; Blue (288–309): transmembrane domain; Red: HA epitope. **B** T-REx HEK cells inducibly expressing SPP (TR-HEK SPP) were transiently transfected with either the C-terminally HA-tagged wild type TREX1 (TREX1-HA) or with the respective SLE-associated TREX1 variants P290L, Y305C and G306A. The cells were treated with 10 μg/mL doxycycline (DOX) for 3,6,9 or 24 h to induce SPP expression. TREX1 processing was assessed subsequently with Western Blotting. **C** Levels of the uncleaved TREX1 precursor were quantified densitometrically from blots as depicted in **B** and normalized to tubulin. The obtained values were then normalized to those of non-induced cells for each mutant individually. Two-Way ANOVA with Dunnett’s post hoc testing. N:3, n = 6. **D** HEK and SPP-deficient (SPP KO) HEK cells were transiently transfected with the SLE-associated variant TREX1-Y305C-HA. Processing of TREX1 was analysed by Western Blotting. **E** After densitometric quantification, total TREX1 band intensities were normalized to those obtained in wild type cells. N:3, n:6. Unpaired two-tailed Student’s t-test. **F** HEK cells were transiently transfected with either wild type (WT) TREX1-HA or with the SLE-associated TREX1 variants also containing a C-terminal HA tag. TREX1 levels and processing were subsequently determined by Western Blotting. **G** Total TREX1 band intensities were normalized to tubulin as loading control. The obtained values were than normalized to the mean ratios obtained for wild type TREX1. N:3, n:6. Unpaired two-tailed Student’s t-test. ns, not significant; ** p ≤ 0.01; *** p ≤ 0.001

Having demonstrated a critical role of the TREX1 TMD for cleavage by SPP, we asked whether any of the reported disease-associated variants in this protein segment may influence proteolysis. This was of particular interest, since several of them involve helix-destabilizing amino acids which are of importance for intramembrane proteases like SPP [39]. We started with the P290L, Y305C, G306A variants (Fig. 4A), which had been linked with SLE [19]. All analyzed variants were targeted to the ER indistinguishably from the TREX1 wild type and co-localized with SPP (Suppl. Fig. 3A). Next, we expressed the respective TREX1 variants in the TR-HEK SPP cell line and performed a time course of SPP induction (Fig. 4B, C). In comparison to the wild type protein, the TREX1 P290L variant was less efficiently cleaved by SPP. Even after 24 h induction, very little reduction of the precursor and transition into the cleavage fragment was seen. In contrast, proteolysis of the TREX1 G306A variant occurred with kinetics comparable to the wild type protein. Assays with the Y305C variant resulted in a prominent double band even in absence of doxycycline (Fig. 4B). SPP induction resulted in a further reduction of the precursor and increase of the cleavage fragment, which occurred with a slightly kinetics as compared to the wild type protein (Fig. 4C). The described findings strongly suggests a constitutive cleavage of the Y305C variant. Upon subcellular fractionation, the cleavage fragment of TREX1 Y305C was detected in the cytosol (Suppl. Figure 3B). As processing occurs already without SPP overexpression, we hypothesized that the Y305C exchange enhances the cleavability of the mutant by endogenous SPP. However, when we expressed this variant in SPP KO cells the cleavage fragment was present in similar amounts as in control cells and still localized to the cytosol (Fig. 4D, Suppl. Fig. 3B). This excludes that SPP is involved in its generation. Nevertheless, since the TREX1 Y305C precursor was slightly enriched in the SPP-deficient cells (Fig. 4D, E), SPP seems to play a role in the turnover of the TREX1 Y305C variant. Similarly, and comparable to the wild type protein, also TREX1 P290L and G306A were slightly stabilized in SPP-deficient cells (Suppl. Fig. 3C–F) confirming that cleavage by endogenous SPP occurs. Finally, we aimed to assess if the altered processing of the three analyzed TREX1 variants had an impact on the steady-state levels of the corresponding proteins. When expressed in HEK cells, the P290L variant was detected with similar abundance as wild type TREX1 (Fig. 4F, G). In contrast, steady-state abundance of the constitutively processed Y305C mutant as well as the G306A variant were reduced, even though rather mildly in the latter case (Fig. 4F, G). In summary, we conclude that the TREX-1 G306G variant did not affect proteolytic processing by SPP, while the P290L and Y305C variant, affect proteolytic processing of TREX1 in a potentially relevant, but opposite way.

### The AGS-causing T303P mutation strongly enhances SPP-mediated degradation of TREX1

**Fig. 5:**
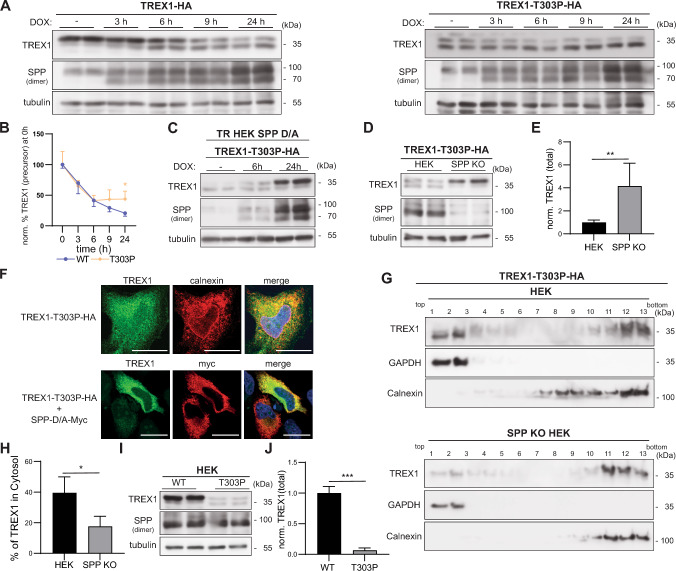
The T303P mutation enhances intramembrane proteolysis of TREX1 by SPP. **A** T-REx HEK cells inducibly expressing SPP (TR-HEK SPP) were transiently transfected with either the C-terminally HA-tagged wild type TREX1 (TREX1-HA) or the mutant TREX1-T303P-HA. The cells were treated with 10 μg/mL doxycycline (DOX) for 3,6,9 or 24 h to induce SPP expression. Subsequently, TREX1 processing was assessed by Western Blotting. **B** Protein levels of the wild type and T303P TREX1 precursor were determined densitometrically and normalized to tubulin. The obtained ratios were then normalized to those of the respective non-induced control. N:3, n:6. Two-Way ANOVA with Dunnett’s post hoc test. **C** T-REx HEK cells inducibly expressing the inactive variant of SPP (TR-HEK SPP D/A) were transiently transfected with the C-terminally HA-tagged TREX1 mutant TREX1-T303P-HA. The cells were treated with 10 μg/mL doxycycline for 6 or 24 h to induce SPP expression. Subsequently, TREX1 processing was assessed by Western Blotting. **D** HEK and SPP-deficient HEK cells (SPP KO) were transiently transfected with TREX1-T303P-HA. TREX1 abundance and processing were analysed by Western Blotting. **E** Band intensities were quantified densitometrically and total TREX1 T303P levels were normalized to that of the corresponding loading control. The obtained rations were then normalized to the mean intensity obtained from WT HEKs. N:3, n:6. Unpaired two-tailed Student’s t-test. **F** TREX1-deficient HeLa cells (ΔTREX HeLa) were transiently transfected with C-terminally HA tagged TREX1-T303P either alone or together with proteolytically inactive SPP (SPP-D/A) carrying a C-terminal Myc epitope. After fixation, cells were analysed by indirect immunofluorescence employing the indicated antibodies. Scale bar, 20 µm. **G** HEK and SPP-deficient (SPP KO) HEK cells transiently expressing TREX1-T303P-HA were subjected to subcellular fractionation. The abundance of the TREX1 precursor and the processed form in fractions containing ER membranes and cytosol was revealed by Western Blotting. **H** The TREX1 T303P amounts in the upper two fractions were normalized to the total TREX1 amount over all gradient fractions. N:3, n:3. Unpaired, two-tailed Student’s t-test. **I** HEK cells were transiently transfected with either wild type TREX1-HA or with the corresponding T303P variant. TREX1 levels and processing were subsequently determined by Western Blotting. **J** Total TREX1 band intensities from blots as depicted in **I** were determined densitometrically and normalized to tubulin as loading control. The obtained ratios were then normalized to those calculated for TREX1-HA. N:3, n:6. Unpaired two-tailed Student’s t-test. * p ≤ 0.05; ** p ≤ 0.01; *** p ≤ 0.001

While the pathophysiological relevance of the P290L, Y305C and G306A variants with regard to the SLE risk is so far mainly correlative, one patient with the T303P variant suffering from AGS has been reported [18]. We describe here two additional patients, siblings, who are homozygous for the TREX1 T303P variant (Suppl. Fig. 4A, B) and presented with AGS in their early childhood (Suppl. Text). Their mother, who is heterozygous for the same TREX1 variant, is asymptomatic. Since the pathogenesis underlying this variant is currently unexplained and it introduces a helix-breaking residue into the TREX1 TMD (Fig. 4A), we reasoned that it could modulate processing by SPP. Therefore, we assessed TREX1 T303P in a SPP co-expression assay in our inducible cell line (Fig. 5A, B). Similar to the Y305C variant, TREX1 T303P was detected as a double band already in non-induced cells, which suggested constitutive cleavage. With increasing SPP expression, the low amounts of the precursor, which were detected, were further depleted, while the cleavage fragment became enriched (Fig. 5A, B). In contrast, upon co-expression of TREX1 T303P with catalytically inactive SPP, a major stabilization of the uncleaved precursor was observed potentially due to protective substrate trapping in the catalytic site of the enzyme (Fig. 5C). We evaluated if SPP contributes to the observed constitutive processing of the T303P mutant by expressing it in SPP KO cells (Fig. 5D, E). Here, the cleavage fragment seen in wild type HEK cells was absent and the abundance of the precursor was strongly increased. Thus, in contrast to TREX1 Y305C, SPP is clearly responsible for the generation of the T303P cleavage fragment. We aimed to determine the subcellular localization of the TREX1 T303P variant. In immunofluorescence analysis, part of the signal was present in the ER, presumably reflecting the uncleaved precursor. However, the additional diffuse staining could point to cytosolic localization of the cleavage fragment (Fig. 5F). This was confirmed by subcellular fractionation of HEK cells with endogenous SPP expression (Fig. 5G, H). Instead, in SPP KO cells, TREX1 T303P was predominantly localized in the ER membrane. Thus, the T303P variant enhances the cleavability of TREX1 by SPP so that the protein is constitutively released into the cytosol. The mutation does not only change the subcellular localization of TREX1, but also its steady-state abundance (Fig. 5I, J). This may indicate, that the half-life of the cleaved form of TREX1 T303P is shorter than that of the membrane-bound precursor so that the overall turnover is accelerated.

**Fig. 6:**
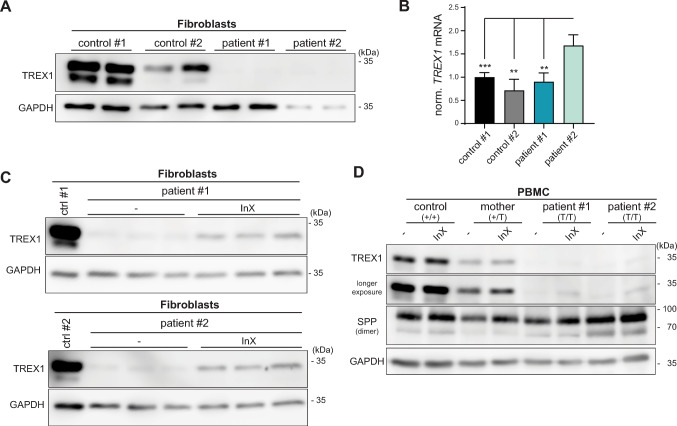
SPP-dependent destabilization of TREX1 in AGS patients with homozygous T303P mutation.** A** Dermal fibroblasts from patients carrying the TREX1 303P mutation as well as two independent commercially obtained control fibroblast cell lines were monitored for their TREX1 levels by Western Blotting. **B** *TREX1* mRNA abundance was quantified in the same cell lines by qPCR. Mean mRNA levels were normalized to those of control #1. N = 2, n = 3(patient #2), n = 4 (rest). One-Way ANOVA with Tukey’s post hoc test. ** p ≤ 0.01, *** p ≤ 0.001. **C** Patient fibroblasts were treated with 1 µM inhibitor X (InX) or DMSO as control for 24 h. TREX1 protein levels were finally compared to those from WT control lines by Western Blotting. **D** PBMCs were isolated from the two patients carrying the TREX1 T303P mutation as well as their mother (heterozygous carrier) or a non-related healthy volunteer. Cells were subsequently treated with DMSO or 1 µM inhibitor X (InX) for 24 h prior to cell lysis. Finally, cellular TREX1 levels were evaluated by Western Blotting

We aimed to investigate, which of our cell line-based findings regarding subcellular localization and protein stability of TREX1 T303P may explain AGS pathogenesis. Therefore, we analyzed primary fibroblasts from the two patients homozygous for TREX T303P in comparison to cells from healthy individuals (Fig. 6A). In the patient cells, the TREX1 protein was hardly detectable. We did not see a reduction of TREX1 mRNA levels in the patient cells, ruling out transcriptional effects as mechanism underlying the TREX1 depletion (Fig. 6B), instead pointing to enhanced degradation of the T303P protein. Therefore, we tested, if inhibition of SPP is able to stabilize the protein (Fig. 6C). In line with our cell line-based results, SPP inhibition increased TREX1 T303P levels. However, only a low reconstitution of TREX1 levels was achieved compared to the wild type controls. These findings were recapitulated in PBMCs (Fig. 6D, Suppl. Figure 5), where a major depletion of TREX1 was observed in the patients’ cells and intermediate TREX1 levels in cells from the heterozygous mother. Again, short-term in vitro inhibition of SPP resulted in a slight stabilization of the TREX1 T303P protein. Our findings confirm a role of SPP in TREX1 degradation and highlight the pathophysiological relevance of TREX1 intramembrane proteolysis.

## Discussion

In this study, we identified TREX1 as novel substrate of SPP, expanding our knowledge about the physiological functions of SPP and TREX1 pathophysiology. The observed effects of the T303P and P290L variants on TREX1 cleavability fit well into our current understanding of SPP proteolysis [39]. Though substrate determinants recognized by SPP and also homologous SPP-like (SPPL) proteases are incompletely understood, helix-destabilizing residues like proline or glycine within the transmembrane domain are a common feature of many substrates of this protease family and also other intramembrane proteases [12, 40–42]. They can increase conformational flexibility of the TMD which facilitates cleavage [43, 44]. Due to its unique geometry, proline is the paradigmatic example of a helix-destabilizing amino acid [45–47]. Consequently, introduction of a proline into the TREX1 TMD would be expected to increase helical flexibility and thus substrate cleavability, which is reflected by our data obtained testing the T303P variant. Along this line, exchange of P290 by a helix-stabilizing leucine accordingly reduced proteolysis. The helix-destabilizing effect of glycine in TMDs is probably weaker than that of proline [46]. Consistently, loss of G306 did not have a detectable impact on TREX1 cleavability by SPP. Nevertheless, in the Bri2 protein, a substrate of the SPP-homologue SPPL2b, glycine residues at specific positions of the TMD were found to be critical for cleavage [41].

The impact of the Y305C exchange on TREX1 is more complex since our results point to a protease other than SPP being responsible for release from the ER membrane. Since the cleavage product has a similar molecular weight like the one generated by SPP from wild type TREX1, a cut either within the TMD or very close to the cytosol-membrane interface can be assumed. The list of known intramembrane proteases accepting substrates with a type II topology beyond SPP is rather short, comprising the different SPPL proteases [3] and site-2 protease (S2P) [48]. Of these, only SPPL2c is resident in the ER [3, 9]. However, since it is exclusively expressed in male germ cells [9], it is unlikely to be involved here. The reported subcellular localizations of SPPL2a, SPPL2b, SPPL3 and S2P are the endo-lysosomal system [49], the plasma membrane [49, 50] and the Golgi apparatus [48], respectively. Whether any of these is responsible for cleaving TREX1 Y305C, remains to be answered as well as the question where the substrate and the protease could meet. Obviously, to reach their destinations these proteases have to pass through the ER. Alternatively, despite no clear Golgi localization of the Y305C mutant was observed (Suppl. Figure 3A) it cannot be excluded that the Y/C exchange facilitates ER exit of TREX1 and its delivery to post-ER compartments. Conspicuously, known S2P substrates exhibit a highly conserved cysteine in their TMD close to the S2P cleavage site [48]. However, experimental evidence that this amino acid is critical for substrate recognition and cleavage by S2P is lacking. Of course, it is also possible that a protease with a cleavage site within the immediate juxtamembrane region, however outside the TMD, is involved.

Our findings provide an explanation how the T303P variant in the transmembrane domain far apart from the DNase domain of TREX1 causes AGS. Enhanced SPP-mediated TREX1 turnover results in a loss-of-function in homozygous individuals. While overexpressed T303P TREX1 was efficiently stabilized in SPP-deficient cells (Fig. 5D, E), we only observed a partial rescue in patient cells upon short term SPP inhibition (Fig. 6C, D). This may be explained by incomplete SPP inhibition by the used compound and a rather slow turnover rate of TREX1 under endogenous conditions. In addition, our data indicate a SPP-independent role of the ubiquitin–proteasome system in degradation of full length TREX1 which may also contribute to the degradation of the T303P mutant. Within this context, it seems likely that components of the ER-associated protein degradation (ERAD) pathway are involved to deliver the membrane form of TREX1 to the proteasome. In this system, the AAA-ATPase p97 can extract integral membrane proteins from the ER [51]. In this context, it is of note that the E3 ubiquitin ligase TRIM24 has been implicated in cancer cells to initiate TREX1 degradation by the proteasome [52]. If p97 and/or SPP are involved in this specific pathway, remains to be investigated.

Regarding the potential pathogenesis mechanisms of the P290L, Y305C and G306A variants, it should be noted that these were identified as risk-increasing alleles for SLE without formal proof that they are part of the disease process [19]. In our hands, their impact on TREX1 was diverse arguing against a unifying mechanism of all three variants. The P290L variant could lead to increased stability of the membrane form of TREX1 while the cytosolic form is less efficiently generated. On the contrary, Y305 is anticipated to significantly increase soluble TREX1 in the cytosol. This is also expected from P212fs or P272fs as well V235fs frameshift mutations resulting in C-terminally truncated proteins, which either represent SLE risk alleles [19] or cause retinal vasculopathy with cerebral leukodystrophy (RVCL) [53]. This indicates that shifting TREX1 from its membrane to the cytosolic form is problematic due to compartment-specific functions of the protein, which are, however, not very well understood so far. Independent of its role as DNase, ER-bound TREX1 was shown to support the function of the oligosaccharyltransferase (OST) complex involved in N-glycan biosynthesis [54]. The DNase domain is present in both forms so that in principle ER-bound as well as soluble TREX1 should be able to clear DNA from the cytosol. Proteolytic liberation into the cytosol increases the mobility of the protein within the cell, which may support this task. It remains to be determined, to which extent membrane-bound and cleaved TREX1 contribute to cytosolic DNA degradation or if there are other pathways which require a soluble form of this protein thereby explaining why TREX1 proteolysis has evolved. On the contrary, soluble TREX1 may also bear a certain risk potential as it was revealed for a RVCL-associated TREX1 frameshift variant, which inhibits homology-directed DNA repair and by this means makes cells more vulnerable to DNA damage [55]. Our results suggest that the intramembrane protease SPP provides a switch between the different forms of TREX1. Though one may assume that SPP cleavage is the lacking mechanism to explain the reported presence of TREX1 in the nucleus [26–30], we did not observe a constitutive nuclear translocation of the cleavage product by immunofluorescence. This could mean that further conditions may need to be fulfilled to initiate its transport into the nucleus such as the induction of DNA damage [26] or cell death.

It should be mentioned that release of a stable cleavage fragment by SPP is not self-evident. While this has also been described for HO-1 in certain contexts [7], the cleavage of several other tail-anchored proteins by SPP results primarily in their degradation [6, 9]. The pathways which determine stability of the cleavage product are not yet very well understood, though the E3 ubiquitin ligases MARCH6 and TRC8 have been implicated in this context [13, 31]. Our results suggest, that SPP has a dual role for TREX1 being a degradative mechanism as well as generating soluble TREX1, which then can be further degraded by the proteasome. It remains to be elucidated what determines the switch between these two pathways and how this is regulated. The TREX1 T303P variant is highly cleaved by SPP, but the cleavage product is obviously rapidly degraded. On the other hand, the cytosolic cleavage product derived from the Y305C variant, which is produced by a different protease, was rather stably detected. This could indicate that the mechanisms determining the fate of soluble TREX1 act on membrane TREX1 in conjunction with SPP. Since the E3 ubiquitin ligase TRIM24 initiates degradation of membrane TREX1, but not of a soluble variant lacking the transmembrane domain [52], it will be important to analyse if this pathway may be linked to SPP. The upregulation of TRIM24 by p53 and the resulting TREX1 degradation facilitates activation of the cGAS/STING pathway in tumour cells and by this means anti-tumour immune responses. Interestingly, SPP is upregulated in several types of cancer [8]. Based on the critical role of TREX1 for determining sensitivity to radiotherapy [56], it will be interesting to analyse how SPP expression in tumours impacts on TREX1 function either by increasing its turnover or by increasing its mobility in the cell. Studying a potential cross-talk with the described p53/TRIM24 pathway [52] and the integration of these mechanisms will be of critical importance.

## Supplementary Information

Below is the link to the electronic supplementary material.

Supplementary file1 (PDF 1899 KB)

## Data Availability

All data are available upon request from the corresponding author.

## Abbreviations

SPP: Signal peptide peptidase
SPPL: Signal peptide peptidase-like
TA: Tail-anchored
AGS: Aicardi-Goutières Syndrome
SLE: Systemic lupus erythematosus
TMD: Transmembrane domain
