# Supplementary material for: The DNase TREX1 is a substrate of the intramembrane protease SPP with implications for disease pathogenesis
Source: Cell Mol Life Sci. 2025 Mar 12;82(1):107. doi: 10.1007/s00018-025-05645-5 (PMC11904002; doi:10.1007/s00018-025-05645-5)
Supplement: Supplementary file 1 — Supplementary file1 (PDF 1899 KB) [file 18_2025_5645_MOESM1_ESM.pdf]

Supplementary Text

Clinical synopsis of AGS patients with homozygous T303P mutation

Patient 1 is a twelve year-old boy, who presented with difficulty achieving developmental milestones in his first months of life. He had initial diminished muscle tone and was diagnosed with cerebral palsy. Microcephaly was noted. Then he developed quadriplegia with spasticity, food refusal and required feeding by G-tube. Brain MRI was reported with global atrophy, white matter volume loss, patchy T2 hyperintensity, no calcifications. Chromosomal microarray was normal. At 4 years of age, he was diagnosed with Aicardi-Goutières syndrome. He developed frequent fever episodes and joint swelling. At age 7, he started to receive ruxolitinib, with reduction of inflammatory episodes. He still presents with randomly occurring episodes of facial and ears flushing, without fever. He has no other skin lesions or rashes and no organomegaly.

Patient 2, sibling of patient 1, is a nine year-old boy who presented at seven months of age, when it was noticed that he was not be able to sit. A few months later, he progressively was able to sit, then stand up and walk, although only a few steps and limited by spasticity. He had microcephaly. He has developmental delay and has not developed speech. He has occasional seizures. A brain MRI showed appropriate myelination and punctate calcifications in the globus pallidus and lentiform nuclei. He does not present with rashes nor organomegaly.

Supplementary Figures

Suppl. Fig. 1

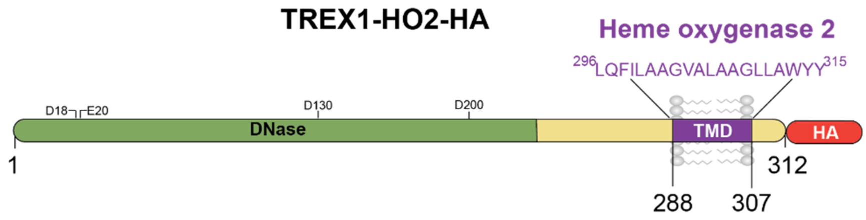

Suppl. Fig. 1. Layout of the TREX1-HO2 chimeric construct. Graphical representation of the protein expressed from the TREX1-HO2-HA construct. Green: part of the protein containing the DNase catalytic domain. According to structural data, the indicated amino acid residues in this section contribute to binding of DNA substrates [25]. Purple: transmembrane domain (TMD) of the HO2 protein (residues 296-315), which were used to replace the TREX1 TMD (288-309); Red: HA epitope.

Suppl. Fig. 2

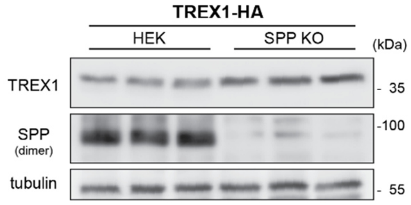

Suppl. Fig. 2. Validation of the SPP KO HEK cell line. C-terminally HA-tagged TREX1-was transiently expressed in HEK and SPP-deficient (SPP KO) HEK cells. The absence of SPP expression in SPP KO cells was confirmed by Western Blotting.

Suppl. Fig. 3

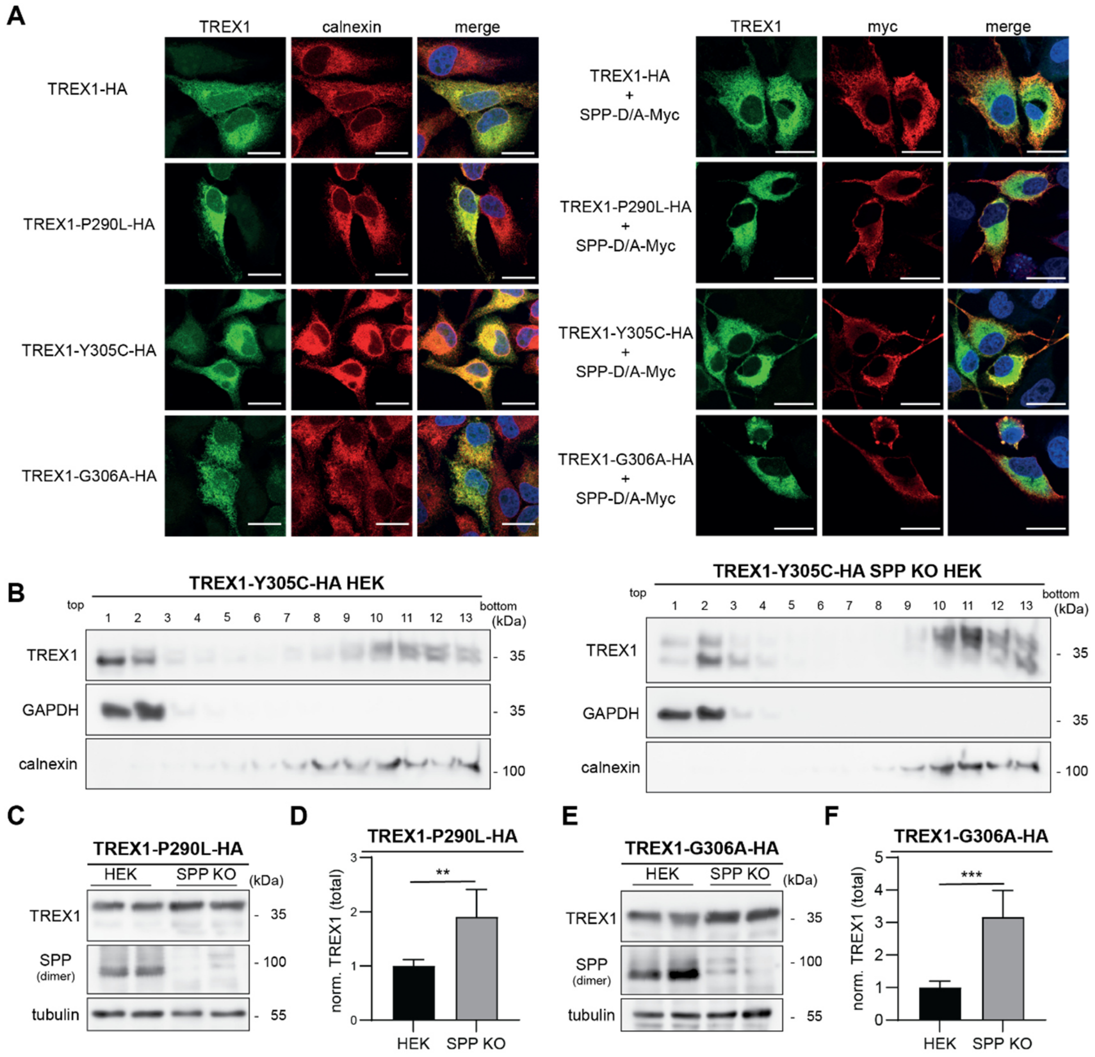

Suppl. Fig. 3. Characterization of the TREX1 variants associated with SLE. A) TREX1-deficient HeLa cells (ΔTREX HeLa) were transiently transfected with expression constructs of C-terminally HA tagged wild type TREX1 or the SLE-associated variants of TREX1-P290L, TREX1-Y305C or TREX1-G306A either alone or together with proteolytically inactive SPP (SPP-D/A) carrying a C-terminal Myc epitope. After fixation, cells were analysed by indirect immunofluorescence employing the indicated antibodies. Calnexin was visualized as ER marker protein. Scale bar, 20 µm. **B)** HEK and SPP-deficient (SPP KO) HEK cells were transiently transfected with the TREX1-Y305C-HA. Following subcellular fractionation, the distribution of TREX1 levels in the gradient fractions was analysed by Western Blotting. **C)** HEK and SPP-deficient (SPP KO) HEK cells were transiently transfected with TREX1-P290L-HA. Processing of TREX1 was analysed by Western Blotting. **D)** For quantification, TREX1 band intensities were determined densitometrically and divided by those of the corresponding loading controls. Afterwards, the obtained ratios were normalized to the mean of the ratios determined for wild type TREX1-HA. Unpaired, two-tailed Student's t-test. ** p≤0.01. **E)** HEK and SPP-deficient (SPP KO) HEK cells were transiently transfected with TREX1-G306A-HA. Processing of TREX1 was analysed by Western Blotting. **F)** For quantification,

TREX1 band intensities were determined densitometrically and divided by those of the corresponding loading controls. Afterwards, the obtained ratios were normalized to the mean of the ratios determined for wild type TREX1-HA. Unpaired, two-tailed Student's t-test. *** $p \leq 0.001$.

Suppl. Fig. 4

A

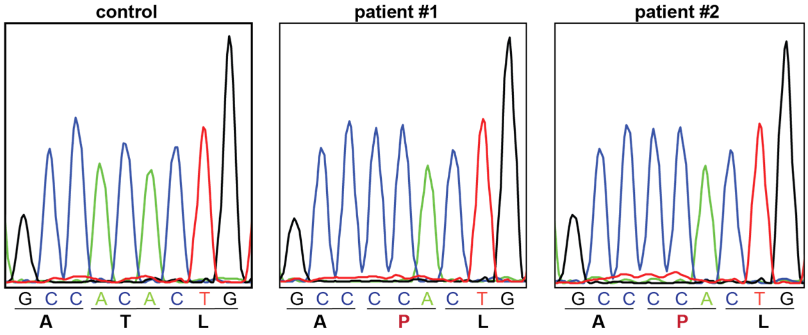

B

```
control       MGSQALPPGPMQTLIFFDMEATGLPFSQPKVTELCLLAVHRCALESPPTSQGPPPTVPPP      60
patient #1    MGSQALPPGPMQTLIFFDMEATGLPFSQPKVTELCLLAVHRCALESPPTSQGPPPTVPPP      60
patient #2    MGSQALPPGPMQTLIFFDMEATGLPFSQPKVTELCLLAVHRCALESPPTSQGPPPTVPPP      60
              ************************************************************

control       PRVVDKLSLCVAPGKACSPAASEITGLSTAVLAAHGRQCFDDNLANLLLAFLRRQPQPWC      120
patient #1    PRVVDKLSLCVAPGKACSPAASEITGLSTAVLAAHGRQCFDDNLANLLLAFLRRQPQPWC      120
patient #2    PRVVDKLSLCVAPGKACSPAASEITGLSTAVLAAHGRQCFDDNLANLLLAFLRRQPQPWC      120
              ************************************************************

control       LVAHNGDRYDFPLLQAELAMLGLTSALDGAFCVDSITALKALERASSPSEHGPRKSYSLG      180
patient #1    LVAHNGDRYDFPLLQAELAMLGLTSALDGAFCVDSITALKALERASSPSEHGPRKSYSLG      180
patient #2    LVAHNGDRYDFPLLQAELAMLGLTSALDGAFCVDSITALKALERASSPSEHGPRKSYSLG      180
              ************************************************************

control       SIYTRLYGQSPPDSHTAEGDVLALLSICQWRPQALLRWVDAHARPFGTIRPMYGVTASAR      240
patient #1    SIYTRLYGQSPPDSHTAEGDVLALLSICQWRPQALLRWVDAHARPFGTIRPMYGVTASAR      240
patient #2    SIYTRLYGQSPPDSHTAEGDVLALLSICQWRPQALLRWVDAHARPFGTIRPMYGVTASAR      240
              ************************************************************

control       TKPRPSAVTTTAHLATTRNTSPSLGESRGTKDLPPVKDPGALSREGLLAPLGLLAILTLA      300
patient #1    TKPRPSAVTTTAHLATTRNTSPSLGESRGTKDLPPVKDPGALSREGLLAPLGLLAILTLA      300
patient #2    TKPRPSAVTTTAHLATTRNTSPSLGESRGTKDLPPVKDPGALSREGLLAPLGLLAILTLA      300
              ************************************************************

control       VATLYGLSLATPGE      314
patient #1    VAPLYGLSLATPGE      314
patient #2    VAPLYGLSLATPGE      314
              ** ***********
```

Suppl. Fig. 4. Genotypic analysis of T303P patient fibroblasts. Genomic DNA was isolated from either wild type or patient fibroblasts carrying the TREX1 T303P mutation. The *TREX1* coding sequence was subsequently amplified by PCR and the corresponding DNA fragments were subjected to Sanger sequencing. The figure displays sequencing chromatograms (A) and predicted protein sequences from a control and the two patients aligned with Clustal Omega Multiple Sequence Alignment.

Suppl. Fig. 5

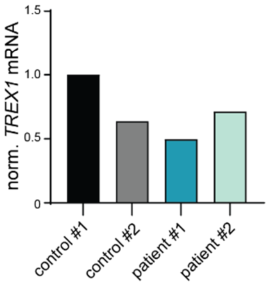

Suppl. Fig. 5. Analysis of TREX1 expression in PBMC. PBMC isolated from donors with the indicated genotypes were monitored for the expression of the *TREX1* gene by qPCR. N=1, n=1 (patient#2) or 2 (rest).
